# The ZT Biopolymer: A Self-Assembling Protein Scaffold for Stem Cell Applications

**DOI:** 10.3390/ijms20174299

**Published:** 2019-09-03

**Authors:** Yevheniia Nesterenko, Christopher J. Hill, Jennifer R. Fleming, Patricia Murray, Olga Mayans

**Affiliations:** 1 Department of Biology, University of Konstanz, 78457 Konstanz, Germany; 2 Department of Cellular and Molecular Physiology, Institute of Translational Medicine, University of Liverpool, Liverpool L69 3BX, UK

**Keywords:** protein design, protein self-assembly, polymer functionalization, biomimetic material, cell culture substrate, stem cells

## Abstract

The development of cell culture systems for the naturalistic propagation, self-renewal and differentiation of cells ex vivo is a high goal of molecular engineering. Despite significant success in recent years, the high cost of up-scaling cultures, the need for xeno-free culture conditions, and the degree of mimicry of the natural extracellular matrix attainable in vitro using designer substrates continue to pose obstacles to the translation of cell-based technologies. In this regard, the ZT biopolymer is a protein-based, stable, scalable, and economical cell substrate of high promise. ZT is based on the naturally occurring assembly of two human proteins: titin-Z1Z2 and telethonin. These protein building blocks are robust scaffolds that can be conveniently functionalized with full-length proteins and bioactive peptidic motifs by genetic manipulation, prior to self-assembly. The polymer is, thereby, fully encodable. Functionalized versions of the ZT polymer have been shown to successfully sustain the long-term culturing of human embryonic stem cells (hESCs), human induced pluripotent stem cells (hiPSCs), and murine mesenchymal stromal cells (mMSCs). Pluripotency of hESCs and hiPSCs was retained for the longest period assayed (4 months). Results point to the large potential of the ZT system for the creation of a modular, pluri-functional biomaterial for cell-based applications.

## 1. Introduction

The study of fundamental processes in cell biology, as well as biomedical and technological cell-based applications, require the culturing of cells ex vivo. For this purpose, culture systems that mimic the native extracellular matrix (ECM) microenvironment by providing biochemical (cell adhesion sites, growth factors) and biophysical (mechanical stiffness) cues are in high demand. Cell culture substrates exist that are derived from natural sources as well as produced synthetically. Prominent examples of naturally derived substrates are Matrigel™ and Geltrex™. The well-studied Matrigel™ is a heterogeneous glycoprotein mixture secreted by mouse sarcoma cells whose composition is comparable to that of an embryonic basement membrane, containing a variety of ECM proteins (particularly laminin-111, collagen IV, entactin, and heparan sulfate proteoglycan) and active growth factors (e.g., fibroblast growth factor, epidermal growth factor, transforming growth factor-β, insulin-like growth factor and platelet-derived growth factor) [1,2]. Although Matrigel™ is widely used for in vitro and in vivo applications [3], it is xenogenic and has a poorly defined, complex composition that suffers from batch-to-batch variability and offers limited experimental control. Simpler functional substrates of better defined composition are constituted by isolated ECM proteins. To this effect, full-length human fibronectin and vitronectin can either be extracted from plasma as native proteins or recombinantly produced (R&D Systems), while laminin isoforms can be expressed recombinantly in human cell lines (BioLamina). However, the production yield of these proteins is limited and their use can be troubled by the presence of impurities, uncontrollable degradation, and, in in vivo applications, possible immunogenicity [4]. Fragments from ECM proteins can also be bioactive, recapitulating the activity of the full-length protein with reasonable efficiency, to the point of supporting the long-term culturing of demanding cell types such as human pluripotent stem cells (hPSCs). Examples of bioactive ECM-protein fragments are: Laminin-511 (a combination of α5, β1 and γ1 laminin chains) produced recombinantly in human embryonic kidney cells [5]; the N-terminal somatomedin B domain of vitronectin expressed in *Escherichia coli* [6]; and the Fn7-Fn14 fragment from fibronectin expressed in mouse myeloma cells [7]. Fragments have the advantage of easing recombinant production and yielding substrates of improved purity. However, they are incomplete mimics of the ECM and proper fragment choice is critical to achieve suitable performance [7].

There is considerable interest in overcoming the limitations of cell substrates based on ECM-components through the engineering of materials that are xeno-free, feeder-free, of controlled composition, and with tailored biological functionalities. To this effect, synthetic substrates have been developed, largely in the form of peptide conjugates [8,9]. A leading example of a synthetic cell substrate is Synthemax™. In this, an acrylate base carries carboxylic acid groups to which short linear peptides (instead of folded proteins) are conjugated using chemical linkers [10]. Synthemax™ fulfills the requirements listed above and successfully supports cell proliferation. However, its usage for long-term cell culturing is not widely spread as some concerns exist related to a possible higher propensity for spontaneous cell differentiation [11] and the potential induction of karyotypic abnormalities [12].

Fulfilling the requirement of improved biodegradability and biocompatibility, materials based on self-assembling peptides have also been developed [13,14,15,16,17]. Peptides self-assemble to form hydrogels and can be designed to be responsive to different physical parameters, like pH and temperature. In addition, peptides produced by chemical synthesis or recombinant technologies offer optimized homogeneity as required to standardize cell-based applications. The molecular design of self-assembling peptides is usually inspired on naturally occurring, self-assembling fibrous proteins, such as collagens, elastin, silk, keratins, amyloids, and coiled-coils. Self-assembling peptides carrying bioactive sequence motifs have yielded excellent achievements in cell-based applications and are now well established [9]. Despite their demonstrated potential, the functionalization limits of peptidic systems in regards to bulky, three-dimensional bioactive components are still uncertain, as the peptidic building block is small and largely invested in mediating the polymeric assembly of the scaffold.

Compared to existing substrates, biomaterials based on self-assembling, full-length proteins can offer economy of production via recombinant methodologies, controlled composition, scalable yields, high purity with low batch variability, biodegradability, full encodability, and ease of “bottom-up” functionalization through genetic engineering—thereby bridging the advantages of synthetic designer peptides and those of natural systems based on ECM-components. Importantly, protein-based materials are ideally suited to exploit modularity by combining multiple protein domains with different functions into a single, multi-block polymer. The prospect of independent tunability of individual domain functions holds high promise for achieving fine control over multiple material properties, which is expected to lead to the development of complex multifunctional matrices of clinical significance [18,19,20]. Yet, designing controlled self-assembly in full-length globular proteins is highly challenging and, currently, designer protein polymers are mostly based on small and well-characterized protein subunits, where a rational molecular design is feasible. A notable example is the consensus tetratricopeptide repeat protein (CTPR), a de novo designed protein system composed of small, independently folded super-secondary helical motifs that can form a range of supramolecular assemblies, including nanofibers, nanotubes, films, and ordered monolayers [21]. Interestingly, strategies for the fusion of symmetric globular proteins and the re-design of proteins that naturally assemble into nano-objects (e.g., viral capsids) are also proving successful, with a variety of assemblies in the form of filaments, molecular layers and 3D-crystals being possible [22,23]. The resulting molecular designs are directed to applications in encapsulation and drug delivery, multivalent epitope display and in synthetic biology. However, such multi-domain protein systems are rarely conceived as microenvironments for cell culturing applications.

We have developed a functionalized, modular biomaterial formed by the controlled self-assembly of two proteins. The polymer, termed ZT, efficiently supports the long-term self-renewal of pluripotent stem cells. Here, we review the molecular design of this new cell substrate, the principles of its assembly and functionalization, and its current application to cell culturing. Its robustness, ease and economy of recombinant production and its high versatility make it a promising system to support complex functionalities in biological applications.

## 2. The ZT Building Block: A Unique Palindromic Assembly of Titin Z1Z2 with its Binding Partner Telethonin

**Figure 1:**
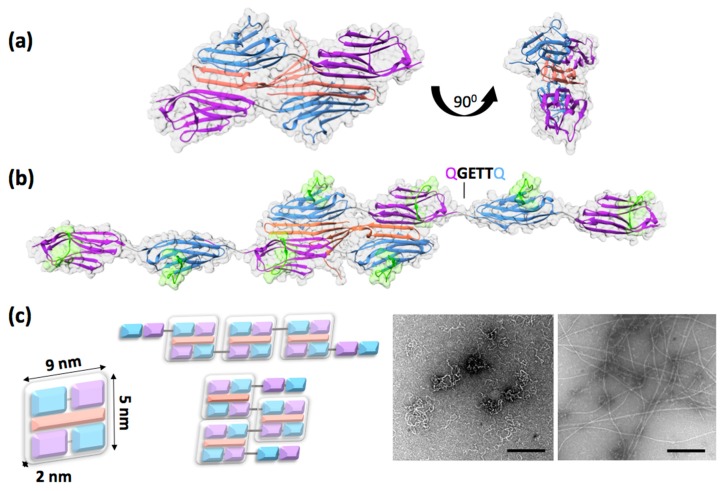
(**a**) Crystal structure of the naturally occurring Z1Z2/Tel complex (Protein Data Bank ID: 1YA5). Domain Z1 is shown in blue, Z2 in purple, and Tel is orange; (**b**) structural model of the Z1Z2-Z1Z2 tandem (Z_1212_) generated by genetically fusing two Z1Z2 pairs using a QGETTQ engineered linker sequence. The model shows the proposed cross-linking of two Z_1212_ units by a Tel molecule at the initial polymerization event. The CD loop serving as internal functionalization site for the grafting of exogenous peptide sequences onto the Z1 and Z2 Ig domains is highlighted in green; (**c**) schematic representation of the Z1Z2/Tel complex with its molecular dimensions (left), its predicted assembly modes (centre), and electron micrographs of the assembled polymer (right). Thin, curly fibers allegedly resulting from sequential tandem assembly can be seen interwoven with thicker, straighter formations possibly corresponding to perpendicular assemblies. Scale bars correspond to 500 nm (left image reproduced from [30] with permission).

The ZT polymer is composed of proteins of human origin produced recombinantly in bacteria. It is based on the naturally occurring complexation of the protein telethonin (Tel) with the two N-terminal immunoglobulin (Ig) domains, Z1Z2, from titin in the Z-disc of the muscle sarcomere. Tel is an insoluble, intrinsically disordered protein that becomes structured upon binding to Z1Z2. Specifically, its 90 N-terminal amino-acid residues become “sandwiched” between two antiparallel Z1Z2 doublets, forming a wing-like hairpin structure that builds an intermolecular β-sheet across the three components (Figure 1a) [24]. The resulting complex is strong and displays a unique palindromic structure. Interestingly, protein complexation through the β-sheet augmentation mechanism is not greatly dependent on sequence as it largely rests on main-chain hydrogen bonding [25,26], a fact that has also been demonstrated for the Z1Z2/Tel association [24]. In this fashion, Tel acts as a sturdy biological glue that joins the N-termini of two titin molecules in the sarcomere.

Z1Z2 and the truncated Tel (residues 1–90) can be expressed recombinantly in bacteria as stable protein products in high yield (>50 mg and >10 mg of pure protein per liter of bacterial culture, respectively). For improved recombinant production, Tel is produced as a Cys-null variant where the five native cysteine residues are exchanged for serines to avoid sample aggregation due to oxidation [27,28]. The Z1Z2/Tel complex displays a high tensile resistance to mechanical forces applied along its molecular axis [29] and its Ig constituent domains are highly thermostable, e.g., the melting temperature of Z1 was 72.6 ± 0.16 °C when monitored using differential scanning fluorimetry [30] and 69.4 ± 0.1 °C when monitored by circular dichroism [31]. Thereby, this two-component protein “sandwich” displayed excellent robustness for biomaterial development.

## 3. Design of the Self-Assembling ZT Polymer

The capability of self-assembling into a high-order polymer was engineered into the Z1Z2/Tel unit by genetically duplicating the coding sequence for Z1Z2 into a double tandem, Z1Z2-Z1Z2 (Z_1212_; Figure 1b). The larger Z_1212_ tandem (41.6 kDa) did not compromise recombinant production in bacterial systems, with high yields being maintained (in excess of 65 mg of pure protein per gram of *E. coli* wet cell mass [30]). The Z_1212_ tandem underwent propagative assembly via sequential Tel-mediated cross-linking [30] and micrographs obtained by transmission electron microscopy (TEM) confirmed the formation of a polymer (Figure 1c). Computational assembly simulations suggested that the observed fibrous formations corresponded to two primary assembly modes: A longitudinal assembly resulting in curly fibers and a transversal assembly leading to a tape-like polymer. Both polymeric formations were thin (the diameter of curly fibers was 7 ± 1.6 nm, while tape-like formations were 13.4 ± 1.6 nm). In routine preparations, the thinner curly formations predominate, being observed with higher frequency and in higher yield.

**Figure 2:**
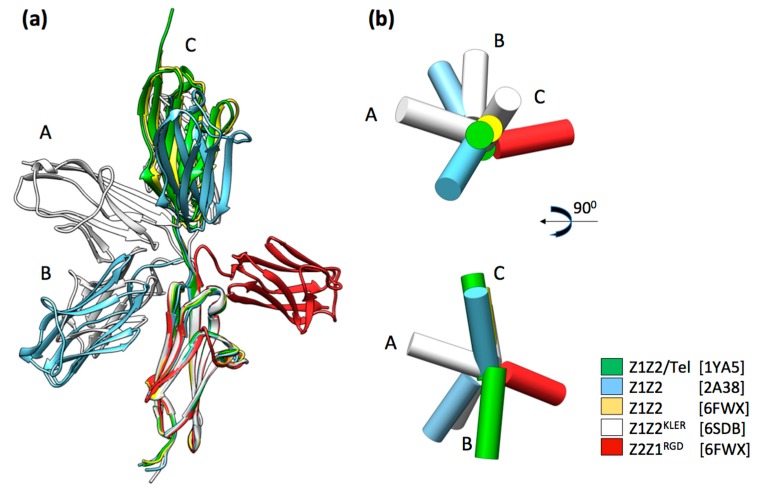
Conformational dynamics in the natural linker sequence of Z1Z2 from titin. (**a**) Conformations of the Z1Z2 Ig-doublets observed experimentally in crystal structures. All pairs are superimposed onto Z1 with the exception of the engineered Z2Z1 pair, where its Z2 domain is superimposed onto Z1 in the other pairs as to allow for comparison. The natural KAET linker sequence that connects Z1 with Z2 is flexible and permits inter-domain motions of large amplitude. In the Z1Z2/Tel pair, the Z1Z2 molecule displayed has been extracted from the structure of the complex illustrated in Figure 1a. The structure of Z1Z2 in isolation (blue) was observed in two conformations: Extended and a tight V-shape. The structure of Z1Z2 carrying the KLER motif in the CD-loop (white) was observed in three inter-domain conformations, here labeled A, B, and C to ease identification. The structure of the Z2–Z1 pair (red) shows that the flexibility of the KAET sequence is reproduced by the engineered linker sequence; (**b**) schematic representation of the domain arrangements shown in (a) where Ig domains are displayed as cylinders (lateral and top views are provided).

The high flexibility and overall morphology of the ZT polymer is determined by the linker engineered in the Z_1212_ fusion tandem: A linker sequence Gln-Gly-Glu-Thr-Thr-Gln (QGETTQ) introduced in the coding DNA between Z1Z2 pairs, which is the only tethering of Z1Z2/Tel blocks in the polymer (Figure 1b,c). In order to avoid the common problem of proteolytically unstable linkers in poly-proteins, this joining sequence was inspired on the Lys-Ala-Glu-Thr (KAET) natural linker between Z1 and Z2 in titin, which was known to be stable. In addition, as polymerization requires Tel to bind sequentially to domain pairs in Z_1212_, the engineered linker was designed to promote extended domain conformations in the tandem Ig. To this effect, the linker included a glutamate residue, which is highly conserved in titin linkers and supports extended domain arrangements through hydrodynamic effects [32,33]. The conformational dynamics of the native KAET hinge in Z1Z2 has been characterized experimentally through the elucidation of the 3D-structure of Z1Z2 using X-ray crystallography and nuclear magnetic resonance (NMR) [34] as well as by analyzing the energetics of hinge closure using molecular dynamics simulations [35,36]. The data showed that the natural Z1Z2 linker was flexible and allowed for a wide range of interdomain conformations (from linearly extended to compact V-shape arrangements) permitting domain motions of large amplitude (Figure 2). The tight V-shape arrangements are short-lived in solution however, as the Z1 and Z2 domains do not form stable direct contacts [34]. The recently available crystal structure of the Z_1212_ tandem (PDB ID: 6FXW) allows comparing the properties of the native (KAET) and engineered (QGETTQ) linker sequences. The structure shows the QGETTQ linker free of interactions and in a bent conformation induced by the tight lattice packing of neighboring domains in the crystal. This indicates that this hydrophilic linker is highly flexible and that it permits a broad range of loose domain conformations, as expected. Further, the high conformational freedom of this linker explains the coiled and very collapsible structure of the polymer, as it constitutes the only junction between adjacent Z1Z2/Tel rigid blocks (Figure 1b,c). Engineering stiffer, extended linkers may allow for the formation of ZT polymers of diverse morphologies. The natural variability of domain junctions in titin could serve as a source of new linkers with defined control on interdomain conformations. Harvesting such natural linkers for polymer design would profit from the existing molecular characterization of titin [37]. Therefore, the linker engineered between Z1Z2/Tel units represents an additional functionalization point of the system, which could be altered to tune the polymer properties, namely its morphology and stiffness.

The loose structure of the ZT polymer appears to facilitate the incorporation and accessibility of bulky functionalization groups in the biomaterial, such as full-length proteins (described in Section 5). Additionally, the avoidance of a compact peptidic aggregate in this case opens the attractive possibility of using ZT as a possibly safe ECM substitute for cells that are destined for in vivo applications. Dense proteolysis-resistant peptidic aggregates (such as amyloidic fibers) cause human disease [38] and the introduction of biomaterials based on dense fibrous aggregates in living systems is a cause for concern. The loose, accessible fibrillar formations of the ZT polymer and the natural existence of the Z1Z2/Tel building block in humans leads to the expected good biodegradability of the material and clearance by the body. Future studies will be required to investigate this aspect.

## 4. Functionalization of the ZT Polymer Using Genetic Engineering: Display of Exogenous Peptide Sequences

The ZT polymer and its components are non-cytotoxic and biologically inert. However, biological functionality can be introduced in the polymer using genetic approaches, such as site-directed mutagenesis or gene fusion, which modify the protein building block pre-assembly. These genetic methods enable the grafting of exogenous peptide sequences onto the ZT scaffold as well as the incorporation of full-length proteins. Contrary to chemical approaches, “bottom-up” engineering provides full control on the amount and stoichiometry of functional groups displayed on the material.

**Figure 3:**
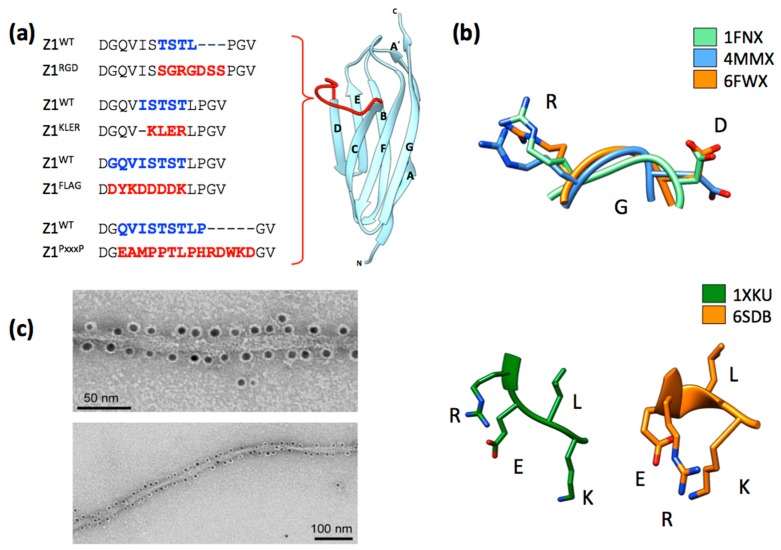
Exogenous peptide display on ZT components. (**a**) Overview of sequences grafted to date onto the CD loop of Z1. The crystal structure of Z1 (extracted from PDB 2A38) is shown and its CD loop is highlighted in red. For each grafted variant, native residues removed from Z1 are indicated in blue and the introduced exogenous residues in red. In the largest substitution, 14 new residues were introduced; (**b**) local structure of the RGD and KLER sequences grafted onto Z1 analyzed using X-ray crystallography and compared to the native sequences in their natural protein environments (only bioactive residues are shown). PDB accession codes for crystal structures are given; (**c**) TEM images of ZT fibers with repetitively attached gold nanoparticles functionalized with Ni^2+^-NTA at different magnifications. The recruitment of the gold nanoparticles (AuNPs) to the ZT polymer was mediated by a His_6_-tag fused N-terminally to Tel. (Images in (c) reproduced from [30] with permission).

The display of peptidic sequences on the ZT polymer is established, with sequences having been introduced both in Tel and Z_1212_ components without preventing polymerization [30,39,40]. Both components permit fusing peptidic sequences to their N- and C-termini and, in addition, Z_1212_ permits the introduction of motifs in four internal positions, namely the CD loop of each of its four Ig domains (Figure 1b). The Ig fold of Z1 and Z2 belongs to the I (intermediate)-type, which lacks developed hyper-variable loops as those of the V (variable)-type that is the archetypal binder fold in nature [41]. Z1 and Z2, as commonly observed in Ig domains from titin, have short loops with partly conserved sequences [32]. However, they possess a surface exposed loop, the CD loop, which is a region of low sequence conservation. We have demonstrated that the CD loop can tolerate drastic diversification in length and composition and have established its suitability as a locus for peptide grafting. Specifically, we replaced the native loop in Z1 for a highly charged FLAG affinity tag (DYKDDDDK) [39], a native PxxP SH3-interaction motif (EAMPPTLPHRDWKD) [39], a sequence carrying the integrin-attachment motif GRGDS from fibronectin (SSGRGDSS) [40], and a decorin-mimetic KLER motif (PDB ID: 6SDB; Figure 3a). All variants could be produced recombinantly in bacteria in yields equivalent to those of the wild-type. The crystal structures of RGD (Z_1212_^RGD^; PDB: 6FWX) and KLER (Z_12_^KLER^; PDB: 6SDB) modified variants as well as the NMR analysis of the FLAG single-domain variant (Z1^FLAG^) [39] confirmed that Z1 retained its native fold and structural integrity after the modifications and that the modified loop did not affect the long-range conformation of the Z_1212_ tandem or its capability to polymerize through interaction with Tel. Furthermore, functional studies demonstrated that FLAG and SSGRGDSS sequences remained accessible and functional upon grafting, respectively supporting molecular and cell-based interactions with good efficiency [39,40]. These results established these Ig domains as successful scaffolds amenable to extensive protein engineering without detriment to their structural integrity, stability or assembly.

The grafting of peptides onto stable protein scaffolds is an established methodology. However, achieving the constrainment of the introduced peptide in its bioactive conformation remains a highly challenging task. The RGD tripeptide is a common cell binding motif present in various ECM proteins including fibronectin, vitronectin, and laminin [42]. Thus, RGD-containing sequences are often grafted on scaffolds designed to support cell attachment [43,44]. However, the conformation of the RGD motif and its direct flanking groups significantly affect integrin selectivity and cell response [44,45]. The crystal structure of Z_1212_^RGD^ proved that the RGD motif incorporated in the CD loop of the third tandem Ig mimicked well the flexibility and the local conformation of the RGD motif within the 10^th^ cell binding domain of native fibronectin, Fn10 (Figure 3b). Size-exclusion chromatography (SEC), SEC-multi angle light scattering, native-polyacrylamide gel electrophoresis (PAGE), and TEM micrographs showed that the introduction of the RGD carrying-sequence at the CD locus did not alter the interaction with Tel or the ensuing polymerization [40]. Subsequent cell assays (below) showed the Z_1212_^RGD^ variant to be functional.

In the Z1Z2 variant carrying the decorin motif KLER in the CD loop of Z1 (Z_12_^KLER^), the motif replaced five native residues so as to become inserted between residues V45 and L51 (Figure 3b). This insertion point was chosen as the V and L residues in Z1 corresponded to the natural flanking residues of KLER in decorin (VKLERL). Decorin is a protein associated with cartilage homeostasis that binds to collagens and plays a role in their stability, organization, and fibrillogenesis [46]. In decorin, the KLER motif is located within a loop of the leucine-rich repeat 3, the region that binds collagen [47] and the incorporation of this sequence in a PEG-peptide co-polymer promoted chondrogenesis of human mesenchymal stromal cells [48]. It was envisioned that the CD loop would constitute a structurally suitable area to incorporate the KLER sequence. The crystal structure of Z_12_^KLER^ (PDB: 6SDB) confirmed that the overall Z1 fold remained intact, but showed that the KLER motif in Z1 (RMSD = 0.34 Å for four non-crystallographic symmetry molecular copies) did not adopt a native conformation as in decorin (PDB: 1XCD). Interestingly, the overall pattern of exposed Lys, Glu, and Arg side chains with a buried Leu sidechain is preserved in Z_12_^KLER^, but the main chain adopts a slight helical turn that causes the side chains to be positioned closer together producing a compacter KLER motif than that of decorin (Figure 3b). Given that loop sequences can undergo changes induced by their binding targets, it remains to be established whether the observed difference affects the biological function of the engineered motif. Nonetheless, a Z_1212_ variant carrying the KLER motif in the CD loop of Z1 domain in third position within the tandem (Z_1212_^KLER^) was created. This was found to maintain the polymerization capability upon mixing with Tel, with its electrophoretic mobility profile being nearly identical to that of wild-type ZT in native-PAGE. In conclusion, data on the various variants establish the suitability of the CD loop as a locus for peptide grafting, capable of supporting the display of bioactive peptidic motifs on the ZT polymer.

Peptide display on the Tel component has also been demonstrated. In this case, polyethylene glycol (PEG)-passivated gold nanoparticles (AuNPs) functionalized with Ni^2+^-NTA were specifically bound to a poly-histidine (His_6_) tag fused to the N-terminus of Tel via an 18 residues linker sequence [30]. The recruitment of AuNPs to the ZT polymer via this affinity tag in Tel resulted in the repetitive decoration of the polymer at the nanoscale (~5 nm inter-particle distance), proving that Tel can display functional and accessible peptides without compromising the polymer structure [30] (Figure 3c).

## 5. Functionalization Through Domain Fusion: Protein Chimeras

**Figure 4:**
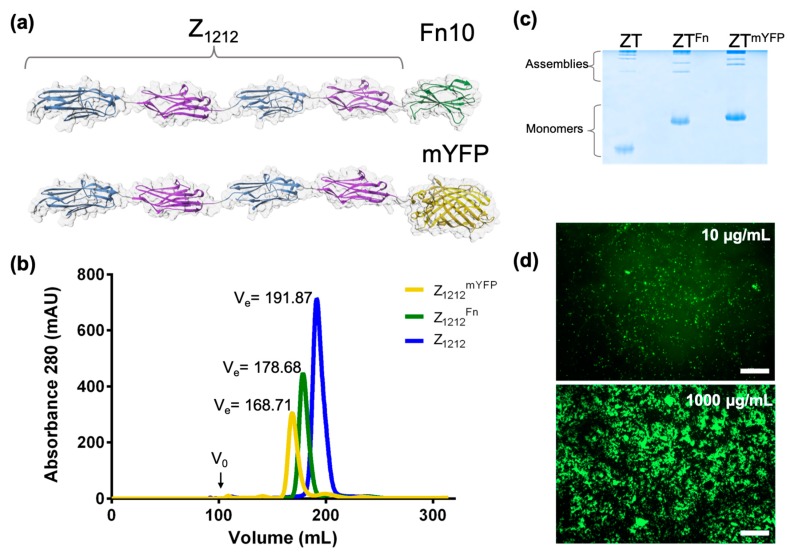
ZT functionalization through genetic fusion of proteins. (**a**) Molecular models of functional chimeric variants of Z_1212_ tandems; (**b**) size exclusion chromatograms of unmodified and chimeric tandems performed on a Superdex S200 26/60 column (GE Healthcare) that demonstrate the monodispersity of the samples (molecular mass: Z_1212_ = 41.76 kDa; Z_1212_^Fn^ = 52.27 kDa; and Z_1212_^mYFP^ = 68.78 kDa); (**c**) native-PAGE of Z-tandems and Tel mixtures 24 h post-assembly; (**d**) fluorescence images of polymeric ZT^mYFP^ adhered onto a polystyrene surface (96 well-plate, COSTAR, Corning) acquired using an Axi Zeiss Zoom V16 fluorescence microscope (Scale bar = 100 µm).

A second approach to the functionalization of the ZT polymer is through the addition of full-length proteins or protein domains to the termini of either Z_1212_ or Tel components via gene fusion. Such incorporations can comprise natural integrin attachment domains, fluorescent proteins, or even processive enzymes. Examples of such functional chimeras are the fusion of the Fn10 domain from fibronectin (Fn10; 10.5 kDa) [40] and the fusion of yellow fluorescent protein (mCitrineFP; 26.9 kDa) to the C-terminus of Z_1212_ (Z_1212_^Fn^ and Z_1212_^mYFP^, respectively; Figure 4a). SEC showed that the chimeric Z_1212_ tandems remained monomeric and highly monodisperse (Figure 4b) [40]. TEM and native-PAGE proved that the chimeric Z_1212_ remained able to polymerize upon complexation with Tel (Figure 4c) [40]. The Z_1212_^mYFP^ chimera polymerized by Tel (ZT^mYFP^) was subsequently used for the coating of polystyrene plates (commonly used in monolayer cell culture) by dispensing on the plastic surface. Fluorescence imaging confirmed that the polymer had adhered to the polystyrene by simple deposition with good homogeneous distribution, demonstrating effective surface coating (Figure 4d). The images revealed that the protein formed concentration-dependent clusters on the polystyrene surface, possibly reflecting the coiled morphology of the polymer. All data considered, we concluded that in the ZT system, the region involved in polymerization is well segregated from the loci of functionalization and that the incorporation of bulky moieties (such as full-length globular proteins) does not disrupt its self-assembly.

## 6. The Functionalized ZT Polymer is an Effective Substratum for Stem Cell Culturing

**Figure 5:**
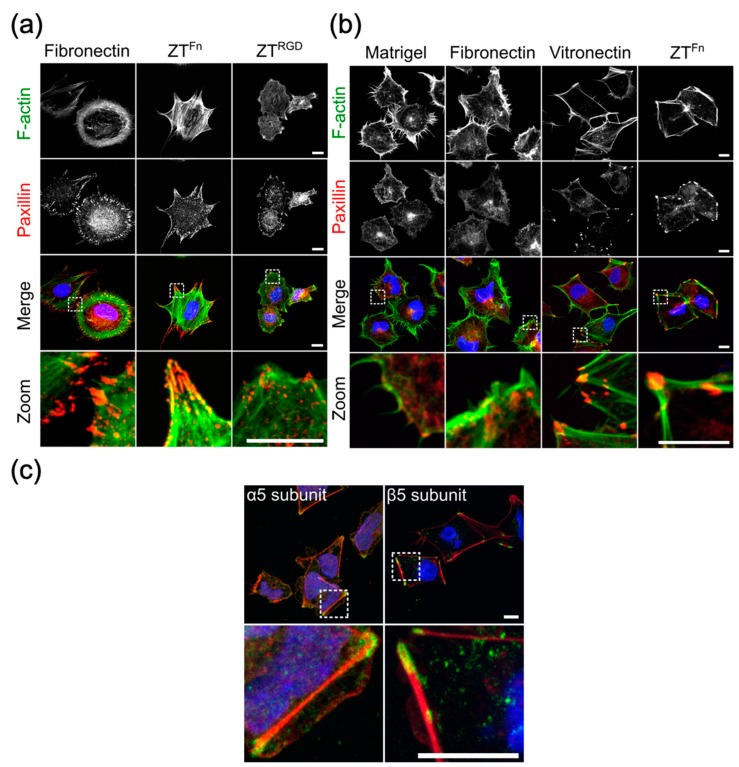
Culturing of murine mesenchymal stromal cells (mMSCs) and human embryonic stem cells (hESCs) on the ZT^Fn^ polymer. Focal adhesion formation and cytoskeletal organization in mMSCs and hESCs cultured on different substrates. Immunofluorescence micrographs show representative z-series projections of mMSCs (**a**) and hESCs (**b**) cultured on ZT variants and control substrates. Cells were stained for F-actin (green), paxillin (red), and DAPI (blue). Zoomed views of the boxed areas in the upper panels are shown to highlight focal adhesions and cytoskeletal structures. (**c**) hESC integrin engagement on ZT^Fn^. Immunofluorescence micrographs show hESCs cultured on ZT^Fn^ and stained for F-actin (red), α5 or β5 integrin subunits (green), and DAPI (blue). Zoomed views of the boxed areas in the upper panels are shown to highlight integrin staining. Scale bars = 10 μm.

**Figure 6:**
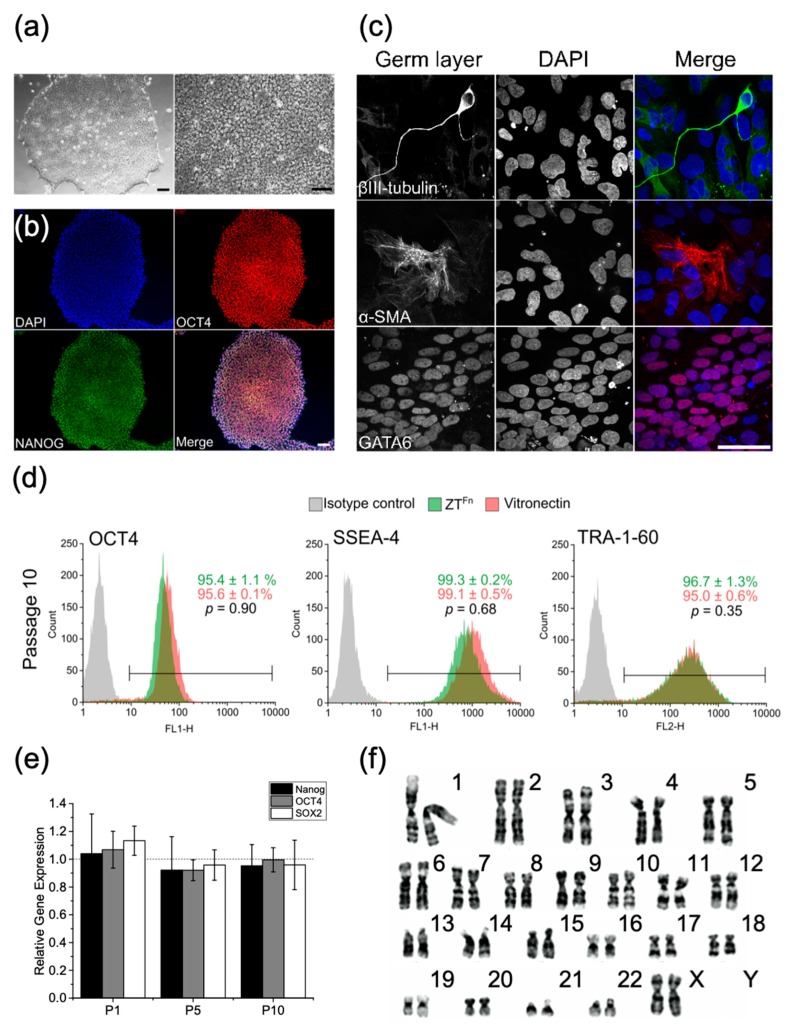
Culturing of human induced pluripotent stem cells (hiPSCs) on the ZT^Fn^ polymer. (**a**) Representative phase contrast micrographs of hiPSCs cultured on ZT^Fn^ for 10 passages. Scale bar = 100 µm. (**b**) Representative epifluorescence micrographs of hiPSCs cultured on ZT^Fn^ for 10 passages. Cells were stained for nuclei (blue), OCT4 (red), and NANOG (green). A merged channel image is shown. Scale bar = 100 µm. (**c**) Confocal micrographs show embryoid body-derived cells stained for markers of the three primary germ layers; β-III tubulin (ectoderm), α-SMA (mesoderm), and GATA-6 (endoderm). Cells were counterstained with DAPI (blue). Scale bar = 50 µm. (**d**) Flow cytometry histograms for pluripotency markers OCT4, SSEA-4, and TRA-1-60 derived from hiPSCs cultured on ZT^Fn^ or vitronectin for 10 passages. The average percentage of positive cells ± SEM is shown (*n* = 3). (**e**) Quantitative RT-qPCR analysis of *NANOG*, *OCT4,* and *SOX2* expression levels in hiPSCs cultured on ZT^Fn^ for 1, 5, and 10 passages relative to cells cultured on vitronectin. Error bars represent SEM (*n* = 3). (**f**) Representative karyogram of hiPSCs cultured on ZT^Fn^ for 10 passages (*n* = 20).

The ZT polymer is commonly produced as a light hydrogel that can be essentially dispensed as a liquid and efficiently adsorbed onto polystyrene surfaces by simple pipetting, acting as a biocoating (Figure 4d). Importantly, the polymer is highly stable in cell culture media and does not become eroded by cell populations. Interestingly, the Ig domains that compose the Z_1212_ tandem have similar molecular dimensions and a related topography to the Fn domains forming native fibronectin. This suggests that the ZT polymer has a coarse molecular granularity that resembles that of components naturally present in the ECM [49]. Upon functionalization by fusion of the Fn10 domain from fibronectin (ZT^Fn^), the polymer has been shown to successfully sustain the long-term culturing of human embryonic stem cells (hESCs), murine mesenchymal stromal cells (mMSCs) [40], and human induced pluripotent stem cells (hiPSCs) [50] (Figure 5 and Figure 6). The ZT^RGD^ polymer variant was shown to elicit mMSC adhesion and spreading, although with lower efficiency than ZT^Fn^ (Figure 5a) [40]. mMSCs are known to be responsive to RGD sequences presented in peptide format [51], but hESCs are more demanding and require more complex substrates with folded ECM components for optimal viability [7]. The hESC line (HUES7) assayed attached and spread on ZT^Fn^ comparably to reference substrates fibronectin, vitronectin, and Matrigel^TM^ (hESCs; Figure 5b) [40]. However, the cytoskeletal structures and cell morphology of cells grown on ZT^Fn^ more closely resembled those cultured on vitronectin. An analysis of integrin engagement upon hESC attachment to ZT^Fn^ showed that αVβ5, an integrin that primarily acts as a vitronectin receptor [52], was engaged along with the canonical fibronectin receptor α5β1 [40] (Figure 5c). This finding adds to early observations, where cells cultured on large fibronectin fragments containing the RGD site were shown to engage preferentially the α5β1 integrin, whereas a small fragment consisting of the Fn10 domain, no longer withheld this preference and bound instead better to the αVβ3 vitronectin receptor [53]. It is now known that αVβ3 and α5β1 are closely related integrins. αVβ3 is somewhat promiscuous and can bind to several ECM proteins including vitronectin, fibronectin, osteopontin, and bone sialoprotein, whilst the α5β1 integrin primarily recognizes fibronectin as a consequence of the presence of the synergistic amino acid sequence PHSRN in the cell attachment site of the protein [54,55]. It must also be considered that RGD motifs are present in many ECM proteins, including fibronectin, vitronectin, fibrinogen, von Willebrand factor, thrombospondin, laminin, entactin, and tenascin, amongst others [42] and that RGD-binding integrin receptors can recognize more than one specific RGD motif [42,52,56]. RGD motifs are individualized by flanking sequence groups as well as unique 3D-structural contexts in their containing proteins, and they often act in unison with other synergy motifs, thereby engaging integrins with differing efficacy. The engagement of prototypical αVβ5 vitronectin integrin receptors by stem cells grown on the fibronectin-based ZT^Fn^ illustrates the plasticity of cell response, where not only the type of bioreactive moiety but also its mode of presentation to the cell and the specific proteomic constitution of a cell type jointly dictate the biological outcome.

In addition to evaluating viability, the long-term stability of stem cells cultured on ZT^Fn^ was assessed by monitoring cell morphology and the expression of transcription factors in hESCs. It was confirmed that the cells retained their embryonic stem cell phenotype, expressing the OCT4 and NANOG transcription factors. Long-term culturing, for up to 10 cell passages proved that hESCs were able to proliferate and maintain a pluripotent phenotype over time, as evidenced by the fact that they could generate embryoid bodies that comprise derivatives of the three embryonic germ layers [40]. These results have been recently extended by our laboratory using cells cultured for 18 cell passages (c.a. 4 months), demonstrating the long-term stability of cells grown on the ZT polymer.

To further validate ZT^Fn^ as a substrate for stem cells, we extended our studies to the culture of hiPSCs [50]. hiPSCs were cultured for up to 10 passages on ZT^Fn^ (under conditions equivalent to those used for the HUES7 hESC line; [40]). Cells retained a canonical PSC morphology typified by colony formation and a high nuclear-cytoplasmic ratio (Figure 6a). Cells also retained nuclear expression of pluripotency markers OCT4 and NANOG (Figure 6b). At the population level, expression of nuclear OCT4 and surface markers SSEA-4 and TRA-1-60 in hiPSCs cultured on ZT^Fn^ for 10 passages were found to be comparable to cells cultured on recombinant vitronectin (from ThermoFisher Scientific) (Figure 6d). Additionally, *OCT4*, *NANOG*, and *SOX2* transcript levels in cells cultured on ZT^Fn^ were not significantly different to those cultured on vitronectin (Figure 6e). Taken together, these data show that ZT^Fn^ is comparable to vitronectin for hiPSC self-renewal. To ensure that culture on ZT^Fn^ did not induce genetic abnormalities, hiPSCs at passage 10 were karyotyped (Cell Guidance Systems). A normal human female karyotype (46, XX) was observed, confirming that the ZT^Fn^ polymer does not induce coarse genetic aberrations (Figure 6f). Finally, an in vitro differentiation assay was employed to confirm maintenance of pluripotency (as previously described for HUES7 cells; [40]). Derivatives of all three germ layers were observed (Figure 6c), demonstrating that hiPSCs remained pluripotent following 10 passages on ZT^Fn^. These results further validate the claim that ZT^Fn^ can be employed for the propagation of hPSCs.

## 7. Conclusions

The need to sustain and study cells ex vivo continues to drive the development of culturing materials that can support a naturalistic cell behavior, thereby, enabling improved research and therapeutic applications. While a variety of cell substrates are now available, few are up-scalable, stable, and economic. Various available systems, like self-assembling peptides (recently reviewed in [9]), short hydrogelation peptides [57,58], and non-peptide based materials such as poly-L-lactic acid (PLLA) and poly-caprolactone (PCL) [59,60] have been employed in cell growth, but their production requires the use of peptide synthesis, harsh solvents (e.g., DMSO, acetone, and dimethylformamide), or methods such as electrospinning. This results in low material yield, higher production cost, and troubles the incorporation of delicate functional proteins. The functionalization of those systems most commonly uses short peptidic motif sequences, which can result in variable or partial efficiency in integrin targeting. When functionalized with full-length proteins, this is mostly done post-production using click chemistry or, in the case of electrospun fibers, oxygen plasma treatment [59,60], which also increases the overall hydrophilicity of the latter material. These functionalization methods, however, allow little control over both the level of incorporation and the distribution of functional moieties on the material, a particularly important aspect when pursuing co-functionalization.

In this regard, we have developed an up-scalable, stable and economical cell substrate based on the naturally occurring palindromic assembly of two proteins of human origin: Z1Z2 and Tel. The robustness, stability, high protein yield, low production cost, and diverse functionalization possibilities in mild media and under biological conditions, are key advantages of the recombinant ZT system in cell-based applications. The assembled ZT polymer is highly flexible and its building blocks act as robust scaffolds that can be functionalized conveniently through the incorporation of bioactive moieties by gene manipulation. Thereby, the polymer is fully encodable and, predictably, biodegradable. The system has been demonstrated to display efficiently both peptidic motifs and full protein domains, with little effect on its polymerization capability. Both Z_1212_ tandem and Tel components can act as recipients of functional moieties at their termini and, in addition, the Z_1212_ tandem contains four internal sites (the CD loops) that can display independent peptidic motifs. Modified building block variants have proven to be stable upon modification and to present a broad tolerance to exogenous sequences. Thereby, the ZT material offers excellent potential for orthogonal functionalization. It is worth noting that in the biomaterial landscape, cell substrates based on large multi-domain proteins of coarse granularity are very scarce. The larger molecular scale of substrates like ZT translates into an enhanced modularity of high promise towards the creation of complex, plurifunctional biomaterials. The conceivable applications of the ZT polymer are diverse, ranging from serving as a scaffold for cell culturing via the incorporation of cell reactive moieties or as a possible drug display system through the incorporation of therapeutic proteins.

## Substrate Availability

Samples of the ZT material can be obtained from the authors on request.

## Abbreviations

AuNPs	Gold nanoparticles
CTPR	Consensus tetratricopeptide repeat protein
ECM	Extracellular matrix
hiPSC	Human induced pluripotent stem cells
hESC	Human embryonic stem cells
Ig	Immunoglobulin
mMSC	Murine mesenchymal stromal cells
NMR	Nuclear magnetic resonance
PAGE	Polyacrylamide gel electrophoresis
PEG	Polyethylene glycol
RGD	Arg-Gln-Asp
SEC	Size-exclusion chromatography
Tel	Telethonin
TEM	Transmission electron microscopy
